# Multifunctionality of silver *closo*-boranes

**DOI:** 10.1038/ncomms15136

**Published:** 2017-04-26

**Authors:** Mark Paskevicius, Bjarne R. S. Hansen, Mathias Jørgensen, Bo Richter, Torben R. Jensen

**Affiliations:** 1 Department of Chemistry and Interdisciplinary Nanoscience Center (iNANO), Aarhus University, Langelandsgade 140, Aarhus C DK-8000, Denmark; 2 Department of Physics and Astronomy and Fuels and Energy Technology Institute, Curtin University, Wark Ave, Bentley Western Australia 6102, Australia

## Abstract

Silver compounds share a rich history in technical applications including photography, catalysis, photocatalysis, cloud seeding and as antimicrobial agents. Here we present a class of silver compounds (Ag_2_B_10_H_10_ and Ag_2_B_12_H_12_) that are semiconductors with a bandgap at 2.3 eV in the green visible light spectrum. The silver boranes have extremely high ion conductivity and dynamic-anion facilitated Ag^+^ migration is suggested based on the structural model. The ion conductivity is enhanced more than two orders of magnitude at room temperature (up to 3.2 mS cm^−1^) by substitution with AgI to form new compounds. Furthermore, the *closo*-boranes show extremely fast silver nano-filament growth when excited by electrons during transmission electron microscope investigations. Ag nano-filaments can also be reabsorbed back into Ag_2_B_12_H_12_. These interesting properties demonstrate the multifunctionality of silver *closo*-boranes and open up avenues in a wide range of fields including photocatalysis, solid state ionics and nano-wire production.

## Abstract (web summary)

Silver compounds have long been known to possess exceptional solid-state conductivity. Here the authors present silver *closo*-boranes in which facile Ag^+^ migration occurs, leading to exceptionally high ion conductivities and potential utility in silver nanowire production and photocatalysis due to their semiconductivity.

Exceptional solid-state conductivity was first noted by Faraday in 1833 for Ag_2_S. He noted that the conducting power was ‘feeble' until heat was applied and it began conducting ‘in the manner of a metal'^1^. Silver iodide is a classic example with the first-order structural transition at 146 °C that results in a step-function increase in Ag^+^ conductivity, 5–6 orders of magnitude higher than at room temperature^2^. AgI is not ideal for technical applications due to poor ion conductivity at room temperature. However, a wide range of silver compounds have been investigated including RbAg_4_I_5_ (ref. 3), which displays one of the highest room temperature solid-state ion conductivities^4^. Silver batteries based on this solid-state electrolyte, Ag|RbAg_4_I_5_|I_2_, show impressive performance even after 20 years of storage^5^. Recently, metal boranes have been identified as highly promising solid state ion conductors^6^. For example, Na_2_B_10_H_10_ and Na_2_B_12_H_12_ have shown high ion conductivities that increase to 0.01 S cm^−1^ above a polymorphic transition temperature (*T*>110 °C), where the *closo*-borane anions undergo rapid dynamic motion and Na^+^ ions can move between partially occupied sites in the crystal structure^7^,^8^,^9^. A large variety of transition metal *closo*-boranes have previously been synthesized (that is, Ag, Cd, Co, Cr, Cu, Fe, Hg, Mn, Ni, Pd, Sc, Zn)^6^, in most cases as solvates due to the strong solvent coordination to the cation. In contrast, the silver cation has a lower charge density and can be isolated as a solvent-free *closo*-borane^10^, but interestingly, only the crystal structures of silver *closo*-borane solvates have previously been described^11^. Silver compounds tend to exhibit another interesting property, the formation of silver filaments, otherwise known as whiskers, capillaries, hair, moss, fibres, wires, spikes, clusters or filiforms. Silver nanowires are interesting in their own right, with potential applications in electronics, photonics, optoelectronics, catalysis and medicine^12^.

Here we present the synthesis, structures and polymorphic transitions of Ag_2_B_10_H_10_ and Ag_2_B_12_H_12_ along with fascinating properties such as semiconductivity, extreme room temperature ion conductivities and metallic filament growth.

## Results

### Structural dynamics

**Figure 1:**
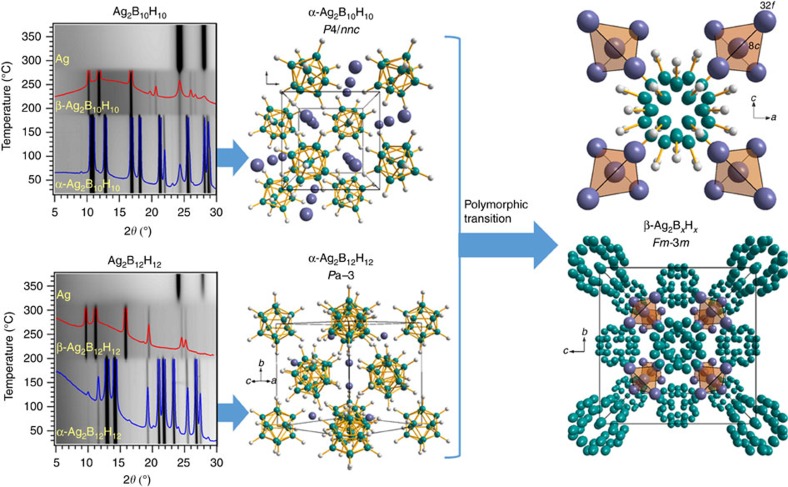
Polymorphic transformations of silver *closo*-boranes. *In-situ* synchrotron radiation powder X-ray diffraction (SR-PXD) data during constant heating at 5 °C per min for Ag_2_B_10_H_10_ and Ag_2_B_12_H_12_ (λ=0.9938 Å). The two low temperature crystal structures (α) are displayed along with their high temperature polymorph (β).

The two silver *closo*-boranes, Ag_2_B_10_H_10_ and Ag_2_B_12_H_12_, both exhibit reversible polymorphic transitions at 180 and 200 °C, respectively, as shown in Fig. 1. The room temperature α-Ag_2_B_10_H_10_ polymorph crystallizes in a tetragonal unit cell in space group *P*4/*nnc*. Ag-atoms are located in the 4*d* Wyckoff site and coordinate tetrahedrally to four B_10_H_10_^2−^ anions with an Ag−H (η^1^) bond length of ∼2.0 Å. The room temperature α-Ag_2_B_12_H_12_ polymorph is cubic (*Pa*-3) and isostructural to α-Li_2_B_12_H_12_ (ref. 13) and Ag_2_B_12_Cl_12_ (ref. 14). The B_12_H_12_^2−^ anions are centred on the faces and corners of the unit cell, while Ag^+^ occupies the 8*c* Wyckoff sites. Each Ag-cation coordinates to three B_12_H_12_-anions with Ag−H (η^2^) bond lengths of ∼2.1–2.4 Å. Further structural details are provided in Table 1, Supplementary Table 1 and Supplementary Figs 1–4.

The crystal structures of the high temperature polymorphs β-Ag_2_B_10_H_10_ and β-Ag_2_B_12_H_12_ are isostructural to one another (*Fm*-3*m*), which is clearly seen from the similarities between the high temperature diffraction patterns in Fig. 1. There is a broad and diffuse X-ray scattering background (∼10°<2*θ*<∼18°) that accompanies the β-polymorphs. This is indicative of rapid ionic motion due to correlations between disordered ions similar to that observed for Ag_2_S (ref. 15), Li_2_B_12_H_12_ and Na_2_B_12_H_12_ (ref. 9), due to cation disorder and/or rapid anion motion. The *closo*-borane anions are structurally described by reoriented partially occupied B−H polyhedra. The structural model extracted from diffraction data cannot differentiate between static or dynamic orientational disorder, but dynamic disorder is well-known in other borane systems from comprehensive studies using nuclear magnetic resonance, quasielastic neutron scattering and neutron vibrational spectroscopy^16^,^17^,^18^.

The silver ions in the β-structures are distributed over two different sites, 32*f* and 8*c*, occupying each crystallographic site equally. The 8*c* site is tetrahedrally surrounded by four 32*f* sites, as shown by the transparent tetrahedra in Fig. 1, but only one of these five sites can be occupied by Ag simultaneously (32*f* site separation ∼2.5 Å compared to the Ag^+^ diameter of 2.58 Å). Each face of the tetrahedron is directed towards an anion superposition and the anions generate an inverted tetrahedron with respect to the Ag-tetrahedron around the 8*c* Wyckoff site. The 8*c* Ag sites permit plausible Ag−H bond lengths (2.0–2.6 Å) for all anion orientations, but are more likely in certain orientations (Supplementary Fig. 5). The 32*f* Ag sites are subjected to a large variability in Ag−H bond lengths (1.4–2.8 Å) depending on the anion orientation. In many cases the 32*f* Ag site has an Ag−H bond length that is physically unrealistic when compared to known Ag−H bond lengths (1.8–2.7 Å) (ref. 11). This suggests that Ag must occupy other crystallographic sites, when the anion is in these orientations, thus supporting the theory of dynamic-anion facilitated Ag^+^ migration, where Ag-ions may be forced to jump from site-to-site by rotating anions.

β-Ag_2_B_10_H_10_ and β-Ag_2_B_12_H_12_ decompose into metallic Ag and a non-crystalline boron-rich compound above 280 and 320 °C, respectively, demonstrating reasonable thermal stability. Interestingly, Ag_2_B_12_H_12_ decomposes endothermically, but Ag_2_B_10_H_10_ decomposes exothermically, suggesting a different decomposition mechanism (Supplementary Figs 6 and 7).

### Ion conductivity

**Figure 2:**
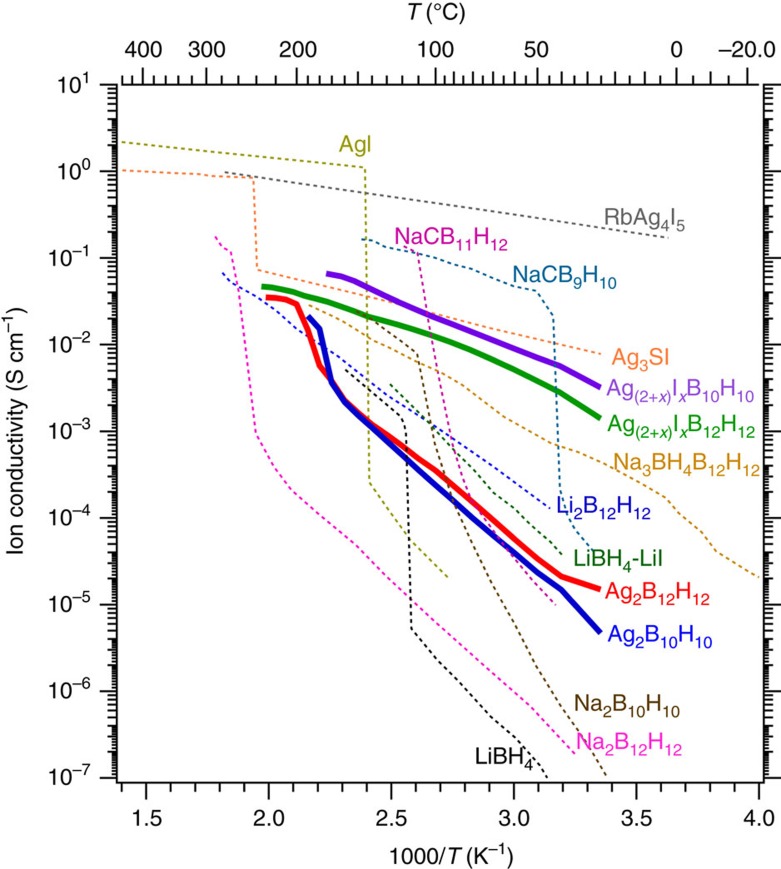
Ion conductivity of the new silver *closo*-boranes. The data (bold) are displayed in comparison to other silver compounds (AgI, RbAg_4_I_5_, Ag_3_SI)^36^ and metal boranes from the literature (Na_2_B_10_H_10_ (ref. 7), Li_2_B_12_H_12_ (ref. 8), Na_2_B_12_H_12_ (ref. 8), NaCB_9_H_10_ (ref. 21), NaCB_11_H_12_ (ref. 20), LiBH_4_ (ref. 37), LiBH_4_-LiI (ref. 22), Na_3_BH_4_B_12_H_12_)^38^.

Two compounds were discovered in the AgI−Ag_2_B_10_H_10_ and AgI−Ag_2_B_12_H_12_ systems, synthesized by annealing 1:1 compositions to study the influence of iodide substitution on ion conductivity. *In situ* SR-PXD of these composites reveals discontinuous changes in Bragg diffraction (Supplementary Figs 8 and 9), which suggest a fixed stoichiometry for new compounds, denoted Ag_(2+*x*)_I_*x*_B_10_H_10_ and Ag_(2+*x*)_I_*x*_B_12_H_12_, possibly with *x*∼1, rather than the formation of solid solutions. Other 1:1 stoichiometric compounds incorporating metal iodides and boranes have been shown previously for *M*_3_IB_12_H_12_ (*M*=NH_4_, K, Rb, Cs)^19^. The Ag^+^ ion conductivity for Ag_2_B_10_H_10_, Ag_2_B_12_H_12_, Ag_(2+*x*)_I_*x*_B_10_H_10_ and Ag_(2+*x*)_I_*x*_B_12_H_12_ are shown in Fig. 2. Ag_2_B_10_H_10_ and Ag_2_B_12_H_12_ have similar ion conductivities and both show a step function in ion conductivity near *T*=180 or 200 °C after the α- to β-polymorphic transition, similar to other metal-B_10_H_10_ or -B_12_H_12_ systems. The new Ag_(2+*x*)_I_*x*_B_10_H_10_ and Ag_(2+*x*)_I_*x*_B_12_H_12_ display vastly improved room temperature ion conductivity in comparison to their parent compounds: AgI, Ag_2_B_10_H_10_ and Ag_2_B_12_H_12_ (Fig. 2). The mixed iodide-boranes form compounds with different structures that improve the room temperature ion conductivity by over two orders of magnitude. At room temperature, the ion conductivity of Ag_(2+*x*)_I_*x*_B_10_H_10_ and Ag_(2+*x*)_I_*x*_B_12_H_12_ are higher than any other metal borane discovered so far (Fig. 2). However, the sodium carboranes (NaCB_9_H_10_ and NaCB_11_H_12_) do exhibit higher ion conductivities at elevated temperature, which can be stabilized to room temperature by quenching, at least temporarily^20^,^21^. Iodide incorporation has also been shown to enhance the ion conductivity of LiBH_4_ by substitution with LiI (Fig. 2) and stabilization of the high temperature polymorph to room temperature^22^.

### Semiconductivity

The silver *closo*-boranes, Ag_2_B_10_H_10_ and Ag_2_B_12_H_12_, display photosensitivity analogous to silver halides, which are well-known for their photoactive properties that have enabled the development of modern photographic film^23^. During synthesis, both materials are initially white powders but become darker in colour over time due to the presence of metallic silver. In fact, the silver *closo*-boranes absorb light over a wide energy band through infrared, visible light and ultra-violet (Supplementary Fig. 10). Ag_2_B_10_H_10_ and Ag_2_B_12_H_12_ both exhibit bandgaps of 2.3 eV (green, 539 nm), lower than that of AgI, 2.8 eV (blue, 443 nm). Thus silver *closo*-boranes provide the possibility to be activated by a larger portion of the solar energy spectrum (∼25%) than ultraviolet based photocatalysts (∼5%). These photoabsorption properties may allow silver *closo*-boranes to act as visible-light photocatalysts. The silver boranes are one of the very few complex hydrides that exhibit semiconductivity, along with the first metal borohydride semiconductor, CsPb(BH_4_)_3_ (ref. 24). A series of Ag-based photocatalysts were recently identified^25^, with Ag_3_PO_4_ demonstrating a quantum yield of nearly 90% (bandgap ∼2.4 eV), performing well in both water splitting and waste-water cleaning applications^26^. Thus, the silver *closo*-boranes may also show promise as future photocatalysts due to their water stability and favourable bandgap.

### Silver nano-filament growth

**Figure 3:**
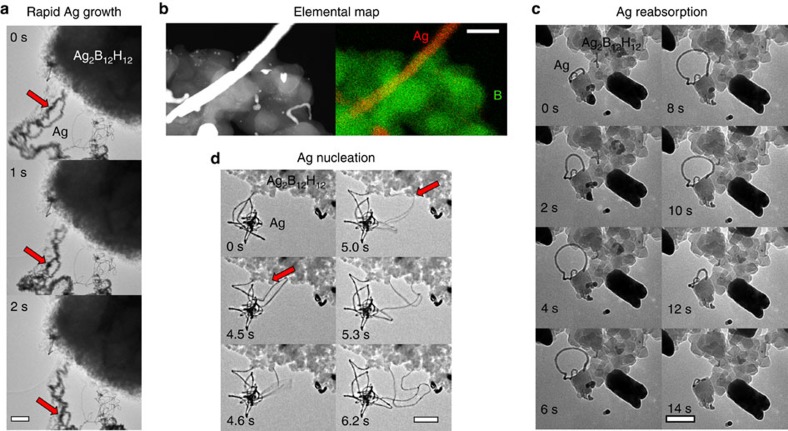
Silver nano-filament growth. Transmission electron microscopy micrographs of Ag_2_B_12_H_12_ demonstrating (**a**) fast Ag growth with an Ag particle moving as shown by the arrow (500 nm scale bar), (**b**) a dark field image and an elemental map showing silver (red) and boron (green) (100 nm scale bar), (**c**) Ag growth and reabsorption (250 nm scale bar) and (**d**) Ag nucleation points created where Ag filaments contact the surface of Ag_2_B_12_H_12_ (see arrows) (100 nm scale bar). Image collection times are shown in seconds. Supplementary Movie 1 shows the dynamics of the process more clearly, collected in real time at 25 frames per second.

The silver *closo*-boranes also undergo rapid electron-driven reduction when imaged by electron microscopy (Fig. 3a and Supplementary Movie 1). Silver filaments (10–50 nm thickness) are rapidly folded on themselves from nucleation points on the surface of the *closo*-borane particle as they are expelled into woven-like bundles of Ag fibres (Fig. 3; Supplementary Fig. 11). The quantity of silver that is expelled is far greater than the local silver content near the Ag_2_B_12_H_12_ nucleation point, demonstrating excellent Ag^+^ conductivity and long-range ion migration from other regions of the particle and possibly through interfaces from other particles. The Ag filament nucleation point appears to allow for the autocatalytic reduction of Ag^+^: if the Ag filament folds back and touches the Ag_2_B_12_H_12_ surface it acts as a second nucleation point, allowing a new filament to form (Fig. 3d and Supplementary Movie 1). In some cases the Ag filaments are also able to be reabsorbed back into the parent Ag_2_B_12_H_12_ particle (Fig. 3c; Supplementary Movie 1). Ag_2_B_12_H_12_ is heavily bombarded by the electron beam during TEM imaging and the high electron current may promote the reduction reaction:


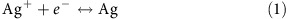


As silver is reduced there will be a negative charge build up due to the isolated B_12_H_12_^2−^ anion. However, the borane anion could also be oxidized to monovalent or neutral species, which have been theoretically and experimentally investigated (that is, B_12_*X*_12_^−^ or B_12_*X*_12_, *X*=H, F, Cl, Br, I)^27^,^28^,^29^.

The Ag formation mechanism may begin in a similar way to the photographic process, where silver atoms are formed at a point within a silver salt when illuminated by photons. These photons promote the formation of electron hole pairs via the photovoltaic effect and a free electron can encounter a lattice defect causing it to become negatively charged, capture Ag^+^ ions, and form Ag (ref. 30). Similarly, free electrons in Ag_2_B_12_H_12_ could promote Ag formation at defect sites. Here, Ag metal can effectively capture and conduct electrons from the charged metallic Ag filament to the Ag_2_B_12_H_12_ contact point, promoting fresh Ag growth. There is limited control over the growth process within the TEM, but an increase in electron current typically initiates or enhances Ag growth, which is still localized to particular nucleation sites. Interestingly, the Ag morphology is always filament-like from these nucleation sites, where silver is drawn from distant regions of the powder. However, the electron-driven process is different to thermal decomposition, where thermally treated Ag_2_B_12_H_12_ shows 50–500 nm Ag particles without filament growth (Supplementary Fig. 12). The study of nanocrystal growth mechanisms is still a developing field, especially with regard to growth mechanisms under an electron beam^31^.

The non-uniform electron beam can induce an electric potential difference across the Ag_2_B_12_H_12_ sample, which could change polarity based on which parts of the particle are electron irradiated, stimulating Ag^+^ migration to balance charge. This behaviour is similar to the controlled growth of Ag from a Ag_2_S scanning tunnelling microscope tip when a voltage is provided, and consequently Ag reabsorption when the voltage polarity is reversed^32^. Because of the electric potential difference in the TEM, we can describe the system shown in Fig. 3c as an Ag|Ag_2_B_12_H_12_|Ag battery, which can drive Ag^+^ from one Ag site to another based on the electron reducing potential at each site. This ion transfer mechanism is the same as in batteries but is instead initiated by the electron beam. *In-situ* analysis of this rapid ion migration may provide new knowledge about electrochemical processes, however because the imaging mechanism is the same as the electron excitation mechanism it is difficult to control. In fact, this is a problem with other battery materials investigated by electron microscopy, where beam irradiation causes the material to undergo chemical and structural evolutions mirroring the charge–discharge cycle^33^.

### Conclusions

The silver boranes presented here show extreme silver mobility in the solid state and a step function in ion conductivity after an α-β polymorphic transition. The high temperature silver *closo*-borane structure reveals a correlation between ideal Ag−H bond lengths and anion orientation. This demonstrates dynamic-anion facilitated Ag^+^ migration, which can be described as a paddle-wheel mechanism for ion conduction if anion reorientation frequencies are found to match cation jump frequencies. These dynamics lead to high ion conductivities, which are enhanced two orders of magnitude at room temperature by forming new compounds with AgI. These materials exhibit the highest room temperature ion conductivities of any metal borane synthesized to date. Anion substitution may prove to be a powerful tool that can be used to tune the crystal structure of ion conductors, leading to order of magnitude increases in ion conductivity. There is still much to learn about the impact that different substituted anions have on the structure and ion conductivity of a material, especially in regard to structural dynamics.

Silver *closo*-boranes may also demonstrate a range of other useful properties. The 2.3 eV bandgap, in the middle of the visible spectrum, and the high stability of Ag_2_B_10_H_10_ and Ag_2_B_12_H_12_ may allow for the design of new photocatalysts in the future. These materials are also stable in water and could lead to new options for photocatalytic water splitting. These results also suggest that it would be promising to investigate the photoactive properties of other silver-based materials with weakly coordinating anions, which could also display useful bandgaps that may follow a trend with anion size. The silver *closo*-boranes display exceptionally fast and reversible silver growth from electron bombardment during TEM imaging on the nanoscale. Thus, the fast silver ion conductors presented here also provide significant insight into nano-growth mechanisms, which mimic battery charging and recharging processes.

## Methods

### Synthesis

(NH_4_)_2_B_10_H_10_ was prepared by a known method from decaborane (Katchem) as follows:^10^


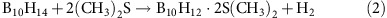



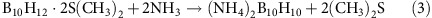


The product was dried under vacuum at 70 °C to remove excess dimethylsulfide and ammonia.

Ag_2_B_10_H_10_ was prepared by adding an aqueous 1 M AgNO_3_ solution dropwise to (H_3_O)_2_B_10_H_10_ in milli-Q water in a 2:1 molar ratio. The acid, (H_3_O)_2_B_10_H_10_, was prepared by passing an aqueous solution of (NH_4_)_2_B_10_H_10_ through an amberlite IR120-H ion exchange resin (Fluka). The solution was stirred and an off-white coloured precipitate, Ag_2_B_10_H_10_, was immediately formed as AgNO_3_ was added. The precipitate quickly darkened in colour over the course of minutes to dark brown as the result of light exposure. The suspension was then filtered and heated to 70 °C under vacuum. The characteristic B−H bending modes of B_10_H_10_^2−^ (2,550 and 2,350 cm^−1^) are clearly observed by FTIR (Supplementary Fig. 13).

Ag_2_B_12_H_12_ was prepared by adding an aqueous 1 M AgNO_3_ solution dropwise to (H_3_O)_2_B_12_H_12_ in milli-Q water in a 2:1 molar ratio. The acid, (H_3_O)_2_B_12_H_12_, was prepared by passing an aqueous solution of Li_2_B_12_H_12_ (Katchem) through an amberlite IR120-H ion exchange resin (Fluka). The solution was stirred and an off-white coloured precipitate, Ag_2_B_12_H_12_, was immediately formed as AgNO_3_ was added. The suspension was then filtered and heated to 70 °C under vacuum. The characteristic B−H bending modes of B_12_H_12_^2−^ (2,450 and 2,320 cm^−1^) are clearly observed by FTIR (Supplementary Fig. 13).

These new silver *closo*-boranes are insoluble in water and can be handled both in water or air without hydrate formation or decomposition, unlike most other metal boranes, which are often hygroscopic or reactive^6^,^34^.

AgI−Ag_2_B_10_H_10_ was hand ground in a 1:1 molar ratio of AgI (Alfa Aesar) and Ag_2_B_10_H_10_ followed by heat treatment under argon to 200 °C for 1 h. *In-situ* SR-PXD data of the formation of Ag_(2+*x*)_I_*x*_B_10_H_10_ (possibly with *x*∼1) during constant heating is shown in Supplementary Fig. 8. A discussion of the crystal structure determination is provided in Supplementary Note 1.

AgI−Ag_2_B_12_H_12_ was hand ground in a 1:1 molar ratio of AgI and Ag_2_B_10_H_10_ followed by heat treatment under argon to 250 °C for 1 h. *In-situ* SR-PXD data of formation of Ag_(2+*x*)_I_*x*_B_12_H_12_ (possibly with *x*∼1) during constant heating is shown in Supplementary Fig. 9. A discussion of the crystal structure determination is provided in Supplementary Note 1.

All chemicals and samples were stored in the absence of light and only handled briefly in light during synthesis and characterization.

### Characterization

*In-situ* synchrotron radiation powder X-ray diffraction (SR-PXD) data were collected at the I711 beamline at MAX II, MAX-lab, Lund, Sweden with λ=0.9938 Å on a Titan CCD 165 mm detector. A hot air blower was used to heat samples at 5 °C per min under argon in a sapphire capillary using a custom made sample cell^35^. SR-PXD data for structure solution were obtained from the P02.1 beamline at Petra III, DESY, Hamburg, Germany at λ=0.20775 Å on a PerkinElmer XRD1621CN3-EHS 410 mm detector. Structure solutions were performed in free objects for crystallography (FOX) and FullProf using rigid body B_10_H_10_^2−^ and B_12_H_12_^2−^ anions.

Electrochemical Impedance Spectroscopy data were collected on a BioLogic MTZ-35 impedance analyser equipped with a high temperature sample holder. Samples were pressed into 6.35 mm diameter pellets of ca. 1 mm thickness and 100 μm gold foil was mechanically fixed to both sides of the pellet, before being placed between platinum electrodes. All measurements were conducted in an argon atmosphere and temperature was measured by a K-type thermocouple 5 mm from the sample. Impedance data were measured at 100 mV ac from 1 to 1 × 10^7^ Hz. Ion conductivity data (*σ*) were derived from Nyquist impedance plots (Supplementary Fig. 14) using the *x*-intercept of the Nyquist semicircle (*I*), area of the pellet face (*A*) and pellet thickness (*t*) according to: *σ*=*t*/(*I* × *A*).

Transmission electron microscopy (TEM) micrographs, Scanning TEM (STEM), and energy dispersive spectroscopy data acquisition was performed on an FEI Talos system equipped with a 200 kV FEG. The STEM/energy dispersive spectroscopy data were collected using the ChemiSTEM system with a High Angle Annular Dark Field (HAADF) detector setup. Rapid TEM micrograph collection was undertaken at 25 frames per second, while collecting 512 × 512 pixel images. Samples were prepared by suspension in toluene and ultrasonication before being dropped onto a holey carbon covered copper grid. The TEM results were reproducible in multiple Ag_2_B_12_H_12_ samples prepared in different batches. Ag_2_B_12_H_12_ exhibited more active Ag growth than Ag_2_B_10_H_10_ and the Ag growth activity was also higher in freshly prepared samples (<4 weeks).

Ultraviolet/VIS data were collected on a Shimadzu ultraviolet-3600 spectrophotometer from 220 to 1,500 nm in 0.5 nm steps. Powders were pressed onto a flat plate BaSO_4_ background in air before analysis. The direct bandgap was calculated using the Kubelka–Munk function with a Tauc plot from 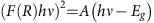, where *hv*=1239.7/*λ* eV, where *λ* is the wavelength in nm, *F*(*R*) is the reflectance spectrum, *A* is a proportional constant and *E*_g_ is the band gap.

FTIR spectra were collected on a NICOLET 380 FT-IR (Thermo Scientific) coupled to a Smart Orbit stage for attenuated total reflectance analysis. The spectra were collected in the wavenumber range of 400–4,000 cm^−1^ with 32 scans.

Differential scanning calorimetry was conducted on a PerkinElmer STA 6,000, where 15–20 mg samples were placed in Al crucibles and heated at 5 °C min^−1^ under constant argon flow (20 ml min^−1^). The instrument was coupled to a Hiden Analytical HPR-20 quadrupole mass spectrometer for residual gas analysis.

### Data availability

The data that support the findings of this study are available from the corresponding author on reasonable request. The X-ray crystallographic coordinates for structures reported in this study have been deposited at the Cambridge Crystallographic Data Centre (CCDC), under deposition numbers CSD-431821—CSD-431824. These data can be obtained free of charge from The Cambridge Crystallographic Data Centre via http://www.ccdc.cam.ac.uk/data_request/cif.

## Additional information

**How to cite this article:** Paskevicius, M. *et al*. Multifunctionality of silver *closo*-boranes. *Nat. Commun.* **8,** 15136 doi: 10.1038/ncomms15136 (2017).

**Publisher's note**: Springer Nature remains neutral with regard to jurisdictional claims in published maps and institutional affiliations.

## Supplementary Material

Supplementary Information Supplementary Figures, Supplementary Table and Supplementary Note

Supplementary Movie 1 Transmission Electron Microscopy (TEM) movie of Ag nanowire growth, nucleation, and absorption from Ag_2_B_12_H_12_.

Peer Review File

## Figures and Tables

**Table 1:** Crystal structure data for silver *closo*-borane polymorphs.

**Chemical formula**	**α**-**Ag**_**2**_**B**_**10**_**H**_**10**_	**β**-**Ag**_**2**_**B**_**10**_**H**_**10**_	**α**-**Ag**_**2**_**B**_**12**_**H**_**12**_	**β**-**Ag**_**2**_**B**_**12**_**H**_**12**_
Crystal system	Tetragonal	Cubic	Cubic	Cubic
Space group	*P*4/*nnc*	*Fm*-3*m*	*Pa*-3	*Fm*-3*m*
*a* (Å)	6.29525(18)	9.6519(4)	9.7654(3)	10.1395(12)
*c* (Å)	10.4330(5)	–	–	–
*V* (Å^3^)	413.46(3)	899.16(6)	931.27(5)	1042.4(2)
*Z*	2	4	4	4
*M* (g mol^−1^)	333.93	333.93	357.56	357.56
*ρ*_calc_ (g ml^−1^)	2.683	2.464	2.531	2.279
*T* (°C)	25	230	25	255

## References

[b1] Faraday M. Experimental researches in electricity. Fourth Series. Philos. Trans. R. Soc. Lond. 123, 507–522 (1833).

[b2] Linford R. G., Pollock J. M. & Randell C. F. Search for new silver-ion solid electrolytes for use in batteries. Nature 259, 656–657 (1976). 1250415 10.1038/259656a0

[b3] Hull S. Superionics: crystal structures and conduction processes. Rep. Prog. Phys. 67, 1233 (2004).

[b4] Hull S., Keen D. A., Sivia D. S. & Berastegui P. Crystal structures and ionic conductivities of ternary derivatives of the silver and copper monohalides: I. Superionic phases of stoichiometry *MA*_4_I_5_:RbAg_4_I_5_, KAg_4_I_5_, and KCu_4_I_5_. J. Sol. State Chem. 165, 363–371 (2002).

[b5] Owens B. B. & Bottelberghe J. R. Twenty year storage test of Ag/RbAg_4_I_5_/I_2_ solid state batteries. Solid State Ionics 62, 243–249 (1993).

[b6] Hansen B. R. S., Paskevicius M., Li H.-W., Akiba E. & Jensen T. R. Metal boranes: progress and applications. Coord. Chem. Rev. 323, 60–70 (2016).

[b7] Udovic T. J. . Exceptional superionic conductivity in disordered sodium Decahydro-closo-decaborate. Adv. Mater. 26, 7622–7626 (2014). 25312377 10.1002/adma.201403157

[b8] He L. . Synthesis of a bimetallic dodecaborate LiNaB_12_H_12_ with outstanding superionic conductivity. Chem. Mater. 27, 5483–5486 (2015).

[b9] Verdal N. . Complex high-temperature phase transitions in Li_2_B_12_H_12_ and Na_2_B_12_H_12_. J. Solid State Chem. 212, 81–91 (2014).

[b10] Muetterties E. L., Balthis J. H., Chia Y. T., Knoth W. H. & Miller H. C. Chemistry of boranes. VIII. Salts and acids of B_10_H_10_^−2^ and B_12_H_12_^−2^. Inorg. Chem. 3, 444–451 (1964).

[b11] Avdeeva V., Malinina E., Sivaev I., Bregadze V. & Kuznetsov N. Silver and copper complexes with closo-polyhedral borane, carborane and metallacarborane anions: synthesis and X-ray structure. Crystals 6, 60 (2016).

[b12] Sun Y. Silver nanowires—unique templates for functional nanostructures. Nanoscale 2, 1626–1642 (2010). 20820692 10.1039/c0nr00258e

[b13] Her J. H. . Crystal structure of Li_2_B_12_H_12_: a possible intermediate species in the decomposition of LiBH_4_. Inorg. Chem. 47, 9757 (2008). 18834192 10.1021/ic801345h

[b14] Tiritiris I. & Schleid T. The crystal structure of solvent-free silver Dodecachloro-closo-dodecaborate Ag_2_[B_12_Cl_12_] from aqueous solution. Z. Anorg. Allgem. Chem. 629, 581–583 (2003).

[b15] Blanton T., Misture S., Dontula N. & Zdzieszynski S. *In situ* high-temperature X-ray diffraction characterization of silver sulfide, Ag_2_S. Powd. Diffr. 26, 114–118 (2011).

[b16] Verdal N. . Evidence of a transition to reorientational disorder in the cubic alkali-metal dodecahydro-closo-dodecaborates. J. Solid State Chem. 184, 3110–3116 (2011).

[b17] Tiritiris I., Schleid T. & Müller K. Solid-State NMR Studies on Ionic closo-Dodecaborates. Appl. Magn. Reson. 32, 459–481 (2007).

[b18] Verdal N., Udovic T. J., Rush J. J., Cappelletti R. L. & Zhou W. Reorientational dynamics of the dodecahydro-closo-dodecaborate anion in Cs_2_B_12_H_12_. J. Phys. Chem. A 115, 2933–2938 (2011). 21413770 10.1021/jp200627f

[b19] Tiritiris I. *Untersuchungen zu Reaktivität, Aufbau und struktureller Dynamik von salzartigen closo*−*Dodekaboraten*, Thesis, Institut für Anorganische Chemie der Universität Stuttgart (2003).

[b20] Tang W. S. . Unparalleled lithium and sodium superionic conduction in solid electrolytes with large monovalent cage-like anions. Energy Env. Sci. 8, 3637–3645 (2015). 26955398 10.1039/C5EE02941D PMC4778258

[b21] Tang W. S. . Liquid-like ionic conduction in solid lithium and sodium monocarba-closo-decaborates near or at room temperature. Adv. Energy Mater. 6, 1502237 (2016).

[b22] Maekawa H. . Halide-stabilized LiBH_4_, a room-temperature lithium fast-ion conductor. J. Am. Chem. Soc. 131, 894–895 (2009). 19119813 10.1021/ja807392k

[b23] Berg W. F. Mechanism of the photographic process. Nature 140, 997–1000 (1937).

[b24] Schouwink P. . Structure and properties of complex hydride perovskite materials. Nat. Commun. 5, 5706 (2014). 25490884 10.1038/ncomms6706

[b25] Li G., Wang Y. & Mao L. Recent progress in highly efficient Ag-based visible-light photocatalysts. RSC Adv. 4, 53649–53661 (2014).

[b26] Yi Z. . An orthophosphate semiconductor with photooxidation properties under visible-light irradiation. Nat. Mater. 9, 559–564 (2010). 20526323 10.1038/nmat2780

[b27] Boeré R. T. . Oxidation of closo-[B_12_Cl_12_]^2−^ to the radical anion [B_12_Cl_12_]^−^ and to neutral B_12_Cl_12_. Angew. Chem. Int. Ed. 50, 549–552 (2011). 10.1002/anie.201004755 21120977

[b28] Morrison J. A. Chemistry of the polyhedral boron halides and the diboron tetrahalides. Chem. Rev. 91, 35–48 (1991).

[b29] Zhao T., Zhou J., Wang Q. & Jena P. Like charges attract? J. Phys. Chem. Lett. 7, 2689–2695 (2016). 27351125 10.1021/acs.jpclett.6b00981

[b30] Greenwood N. N. & Earnshaw A. Chemistry of the Elements Elsevier (1984).

[b31] Longo E., Avansi W., Bettini J., Andrés J. & Gracia L. *In situ* transmission electron microscopy observation of Ag nanocrystal evolution by surfactant free electron-driven synthesis. Sci. Rep. 6, 21498 (2016). 26979671 10.1038/srep21498 PMC4793220

[b32] Terabe K., Nakayama T., Hasegawa T. & Aono M. Formation and disappearance of a nanoscale silver cluster realized by solid electrochemical reaction. J. Appl. Phys. 91, 10110–10114 (2002).

[b33] Lin F., Markus I. M., Doeff M. M. & Xin H. L. Chemical and structural stability of lithium-ion battery electrode materials under electron beam. Sci. Rep. 4, 5694 (2014). 25027190 10.1038/srep05694 PMC4100024

[b34] Pitt M. P., Paskevicius M., Brown D. H., Sheppard D. A. & Buckley C. E. Thermal stability of Li_2_B_12_H_12_ and its role in the decomposition of LiBH_4_. J. Am. Chem. Soc. 135, 6930–6941 (2013). 23581497 10.1021/ja400131b

[b35] Hansen B. R. S. . *In situ* X-ray diffraction environments for high-pressure reactions. J. Appl. Crystallogr. 48, 1234–1241 (2015).

[b36] Rickert H. Solid ionic conductors: principles and applications. Angew. Chem. Int. Ed. 17, 37–46 (1978).

[b37] Udovic T. J. . Sodium superionic conduction in Na_2_B_12_H_12_. Chem. Commun. 50, 3750–3752 (2014). 10.1039/c3cc49805k 24584582

[b38] Sadikin Y., Brighi M., Schouwink P. & Černý R. Superionic conduction of sodium and lithium in anion-mixed hydroborates Na_3_BH_4_B_12_H_12_ and (Li_0.7_Na_0.3_)_3_BH_4_B_12_H_12_. Adv. Energy Mater. 5, 1501016 (2015).

