# Supplementary material for: Multifunctionality of silver closo-boranes
Source: Nat Commun. 2017 Apr 26;8:15136. doi: 10.1038/ncomms15136 (PMC5414069; doi:10.1038/ncomms15136)
Supplement: Supplementary Information — Supplementary Figures, Supplementary Table and Supplementary Note [file ncomms15136-s1.pdf]

Supplementary Information

Supplementary Table 1. Atomic details for the crystal structures of silver *closo*-boranes. See Table 1 for further details.

	Wyckoff	*x*	*y*	*z*	*U* / Å^2	Occ.
α–$Ag_2B_{10}H_{10}$						
Ag	4*d*	0.250	0.750	0.000	0.0702	1
B1	16*k*	0.338	0.434	0.826	0.00362	1
B2	4*e*	0.250	0.250	0.925	0.00362	1
H1	16*k*	0.407	0.591	0.862	0.0380	1
H2	4*e*	0.250	0.250	1.034	0.0380	1
β–$Ag_2B_{10}H_{10}$						
Ag1	8*c*	0.250	0.250	0.250	0.250	0.374
Ag2	32*f*	0.161	0.161	0.661	0.179	0.157
B1	24*e*	0.806	0.000	0.000	0.0981	1/3
B2	96*k*	0.935	0.881	0.935	0.0981	1/3
H1	24*e*	0.689	0.000	0.000	0.0380	1/3
H2	96*k*	0.895	0.780	0.895	0.0380	1/3
α–$Ag_2B_{12}H_{12}$						
Ag	8*c*	0.624	0.624	0.624	0.0564	1
B1	24*d*	-0.087	-0.094	0.125	0.0349	1
B2	24*d*	-0.033	-0.043	-0.163	0.0349	1
H1	24*d*	-0.152	-0.158	0.212	0.0380	1
H2	24*d*	-0.059	-0.071	-0.283	0.0380	1
β–$Ag_2B_{12}H_{12}$						
Ag1	8*c*	0.250	0.250	0.250	0.204	0.521
Ag2	32*f*	0.161	0.161	0.661	0.145	0.120
B1	96*k*	0.445	0.445	0.857	0.0473	1/4
B2	48*h*	0.500	0.622	0.878	0.0473	1/2
H1	96*k*	0.411	0.411	0.753	0.0380	1/4
H2	48*h*	0.500	0.304	0.804	0.0380	1/2

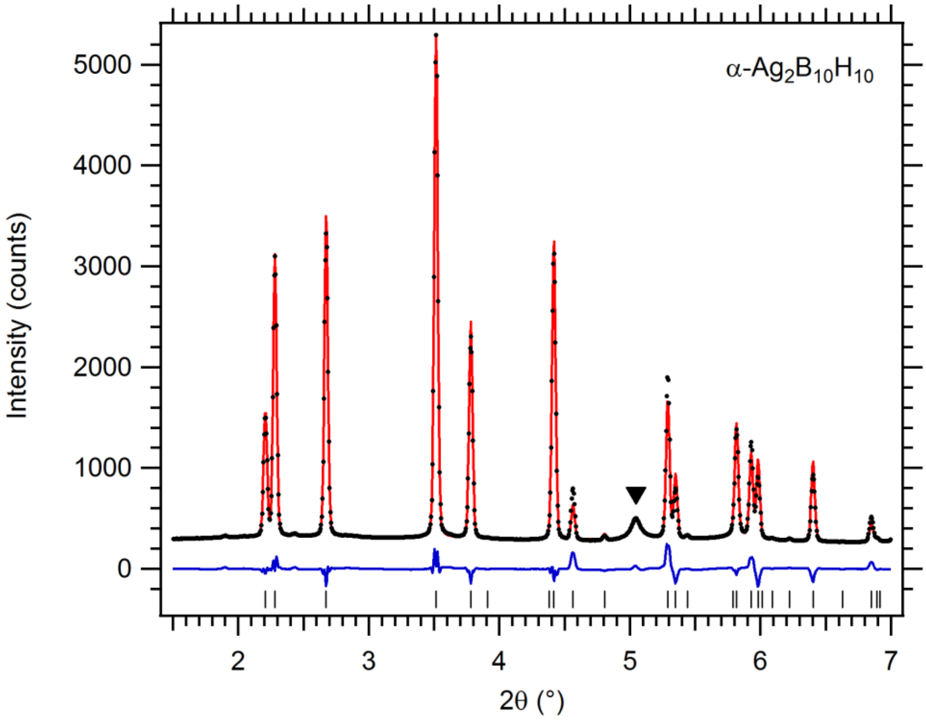

Supplementary Figure 1. Powder X-ray diffraction pattern of α–$Ag_2B_{10}H_{10}$ including Rietveld refinement profile (25 °C). The raw data (black dots), calculated pattern (red line), difference plot (blue line), and *hkl* ticks (vertical lines) are all shown. The black inverted triangle indicates the position of Ag metal. The wavelength is λ = 0.20775 Å and agreement factors are χ^2 = 7813, R_{wp} = 6.02, R_{bragg} = 6.64.

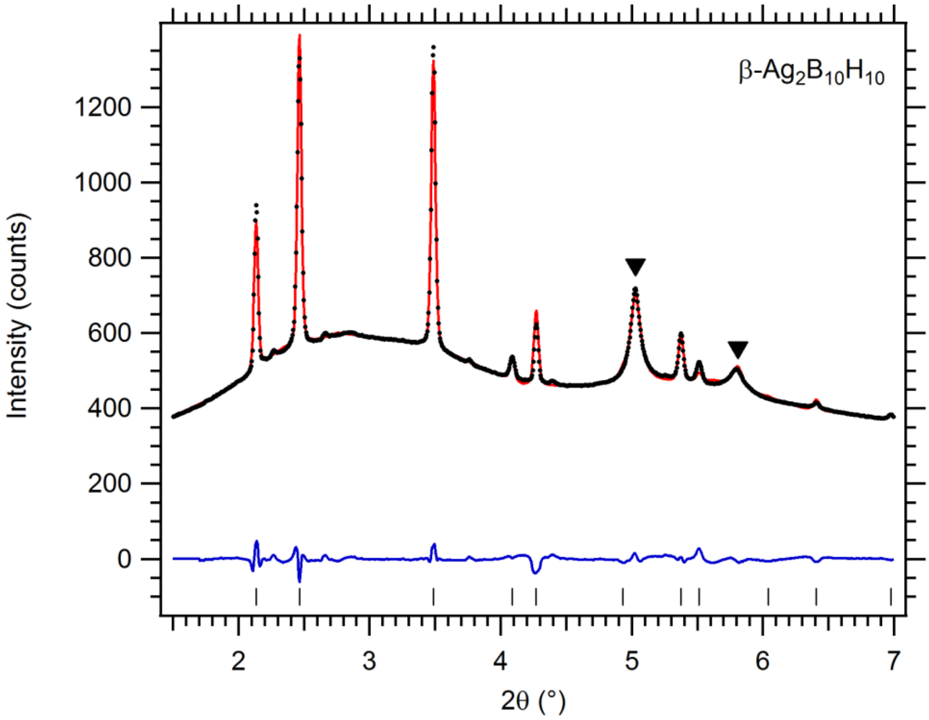

Supplementary Figure 2. Powder X-ray diffraction pattern of β–$Ag_2B_{10}H_{10}$ including Rietveld refinement profile (230 °C). The raw data (black dots), calculated pattern (red line), difference plot (blue line), and *hkl* ticks (vertical lines) are all shown. The black inverted triangles indicate the position of Ag metal. The wavelength is λ = 0.20775 Å and agreement factors are χ^2 = 313.6, R_{wp} = 1.01, R_{bragg} = 3.02.

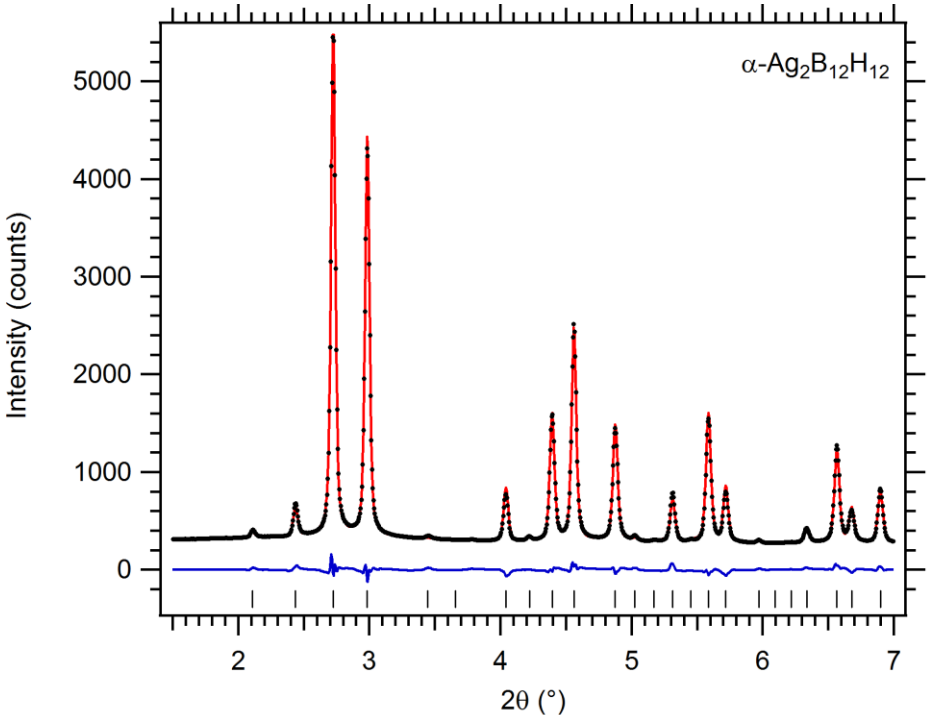

Supplementary Figure 3. Powder X-ray diffraction pattern of α–$Ag_2B_{12}H_{12}$ including Rietveld refinement profile (25 °C). The raw data (black dots), calculated pattern (red line), difference plot (blue line), and *hkl* ticks (vertical lines) are all shown. The wavelength is $\lambda = 0.20775$ Å and agreement factors are $\chi^2 = 1742$, $R_{wp} = 2.63$, $R_{bragg} = 3.12$.

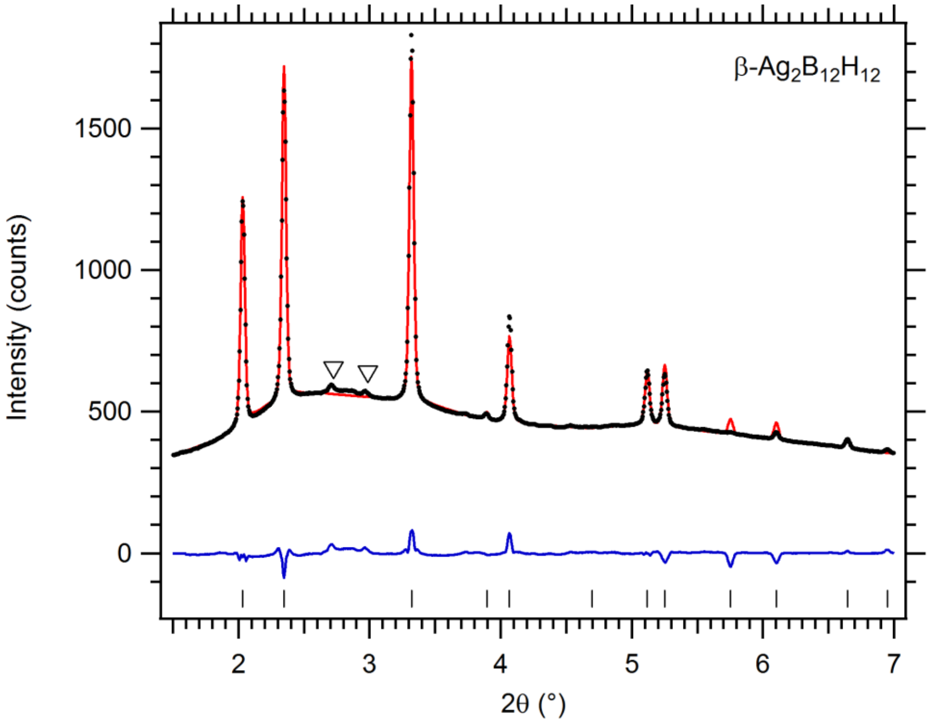

Supplementary Figure 4. Powder X-ray diffraction pattern of β–$Ag_2B_{12}H_{12}$ including Rietveld refinement profile (255 °C). The raw data (black dots), calculated pattern (red line), difference plot (blue line), and *hkl* ticks (vertical lines) are all shown. The open inverted triangles indicate the position of residual α–$Ag_2B_{12}H_{12}$. The wavelength is $\lambda = 0.20775$ Å and agreement factors are $\chi^2 = 578.3$, $R_{wp} = 1.54$, $R_{bragg} = 2.28$.

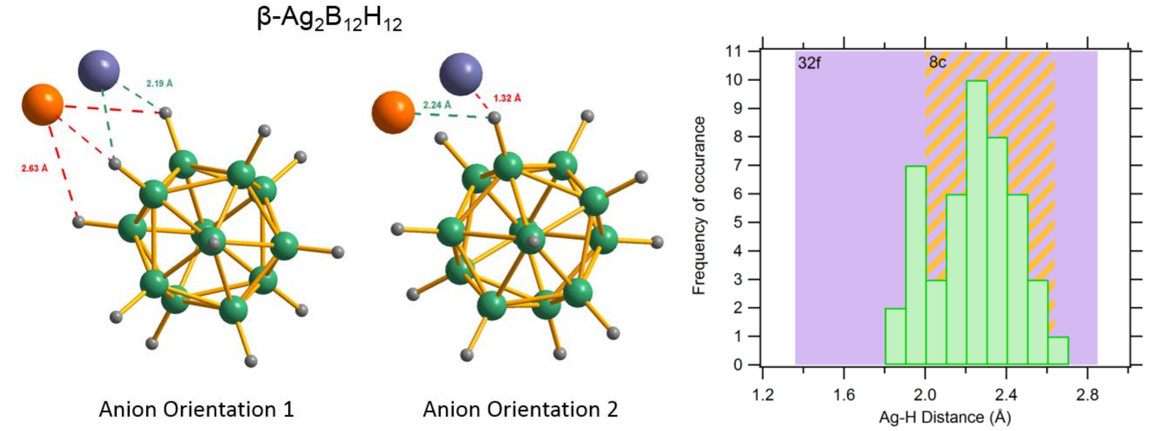

Supplementary Figure 5. $B_{12}H_{12}^{2-}$ anion orientations in β–$Ag_2B_{12}H_{12}$. The 32*f* (purple) and 8*c* (orange) Ag sites are only available to be occupied for particular anion orientations due to limiting Ag–H distances in certain orientations. The Ag–H distance should be ca. 2.3 Å based on the known ambient silver *closo*-borane structures and a histogram (green) of known Ag–H distance for a variety of Ag boranes is shown, derived from [V. Avdeeva, E. Malinina, I. Sivaev, V. Bregadze and N. Kuznetsov, Crystals, 2016, 6, 60]. The 8*c* Ag sites (orange region) permit plausible Ag–H bond lengths for all orientations, but are more likely in certain anion orientations. The 32*f* Ag sites (purple region) are subjected to a large variability in Ag–H bond lengths depending on the anion orientation. In many cases the 32*f* Ag site has an Ag–H bond length that is physically unrealistic, suggesting that the Ag must occupy other crystallographic sites when the anion is in these orientations, thus supporting the theory of dynamic-anion facilitated Ag^+ migration.

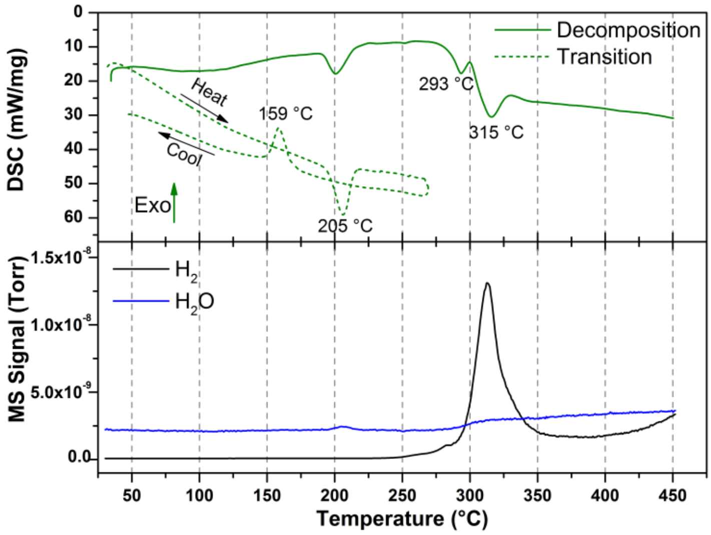

Supplementary Figure 6. Differential scanning calorimetry (DSC) and mass spectroscopy (MS) data of $Ag_2B_{12}H_{12}$. Two DSC measurements are shown, one illustrating decomposition (solid line) and another showing the reversible polymorphic phase transformation (dashed line).

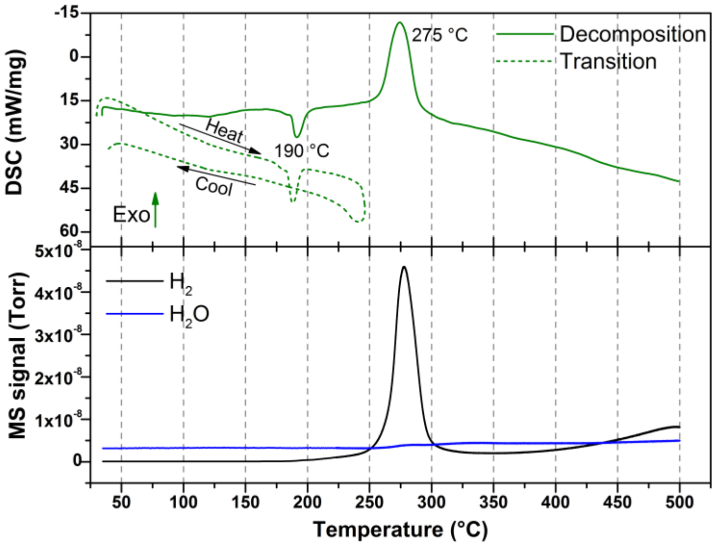

Supplementary Figure 7. Differential scanning calorimetry (DSC) and mass spectroscopy (MS) data of $Ag_2B_{10}H_{10}$. Two DSC measurements are shown, one illustrating decomposition (solid line) and another showing the polymorphic phase transformation (dashed line). In this case, the polymorphic phase transition appears to be sluggish and does not immediately change back to the β–polymorph on cooling.

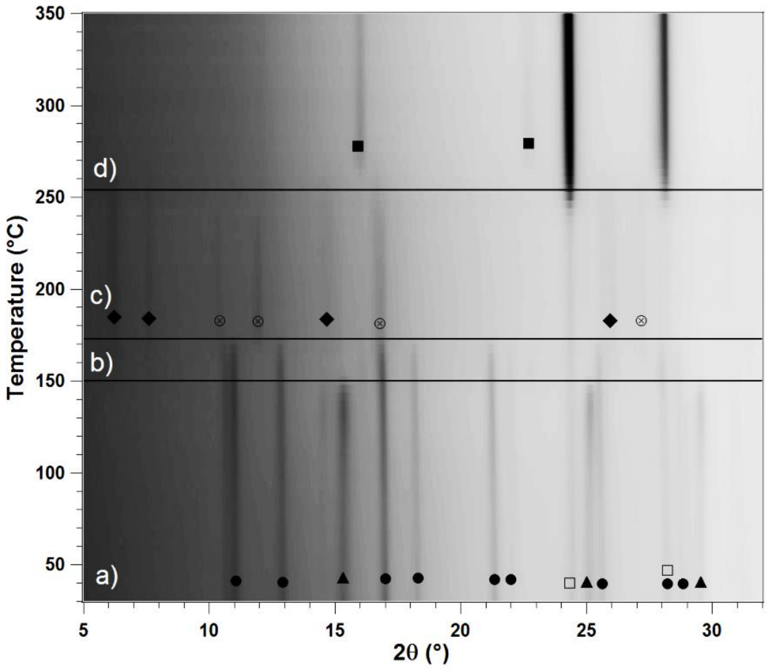

Supplementary Figure 8. *In-situ* synchrotron radiation powder X-ray diffraction (SR-PXD) data of a hand ground 1:1 molar mixture of $Ag_2B_{10}H_{10}$ and AgI ($\Delta T/\Delta t$ = 10 °C/min, λ = 0.9938 Å). Markers: α–$Ag_2B_{10}H_{10}$ (black circle), γ–AgI (black triangle), Ag (open square), β–$Ag_2B_{10}H_{10}$ (open circle), α–AgI (black square), AgI–$Ag_2B_{10}H_{10}$ (black diamond). A) After hand grinding AgI is found in the γ-polymorph at room temperature. B) At 150 °C crystalline γ–AgI disappears but rather than forming α–AgI it appears to be incorporated into the α–$Ag_2B_{10}H_{10}$ structure. C) At 170 °C α–$Ag_2B_{10}H_{10}$ transforms into β–$Ag_2B_{10}H_{10}$ and a new compound denoted $Ag_{(2+x)}I_xB_{10}H_{10}$ (possibly with x ~ 1). D) Upon decomposition, crystalline Ag is formed along with α-AgI, which is released from $Ag_{(2+x)}I_xB_{10}H_{10}$.

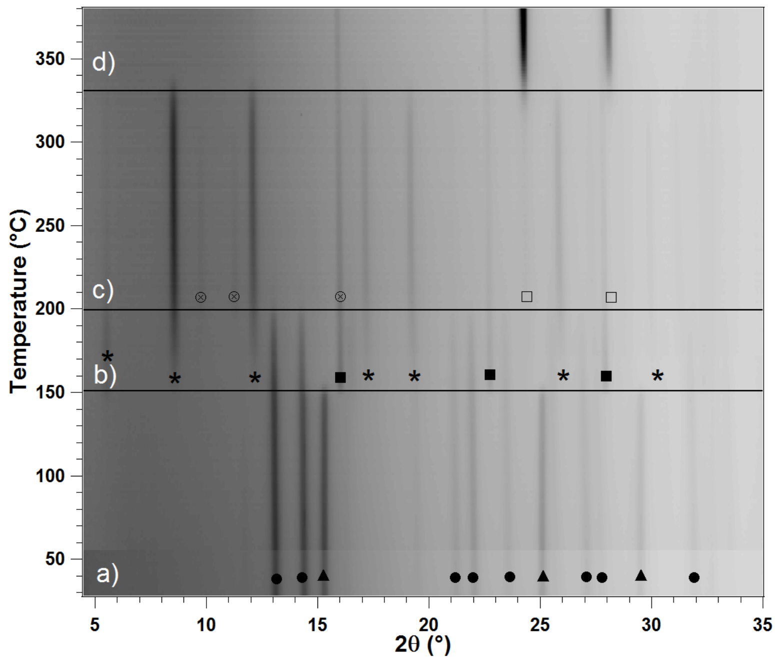

Supplementary Figure 9. *In-situ* synchrotron radiation powder X-ray diffraction (SR-PXD) data of a hand ground 1:1 molar mixture of $Ag_2B_{12}H_{12}$ and AgI ($\Delta T/\Delta t$ = 5 °C/min, λ = 0.9938 Å). Markers: α–$Ag_2B_{12}H_{12}$ (black circle), γ–AgI (black triangle), Ag (open square), β–$Ag_2B_{12}H_{12}$ (open circle), α–AgI (black square), AgI–$Ag_2B_{12}H_{12}$ (asterisk). A) A mixture of α–$Ag_2B_{12}H_{12}$ and γ–AgI is found at room temperature. B) At 150 °C a small portion of γ–AgI transforms into α–AgI and a new compound denoted $Ag_{(2+x)}I_xB_{12}H_{12}$ (possibly with x ~ 1) slowly forms as α-$Ag_2B_{12}H_{12}$ is consumed. C) at 200 °C a residual quantity of α–$Ag_2B_{12}H_{12}$ transforms to β–$Ag_2B_{12}H_{12}$. D) Ag is formed upon decomposition of $Ag_{(2+x)}I_xB_{12}H_{12}$.

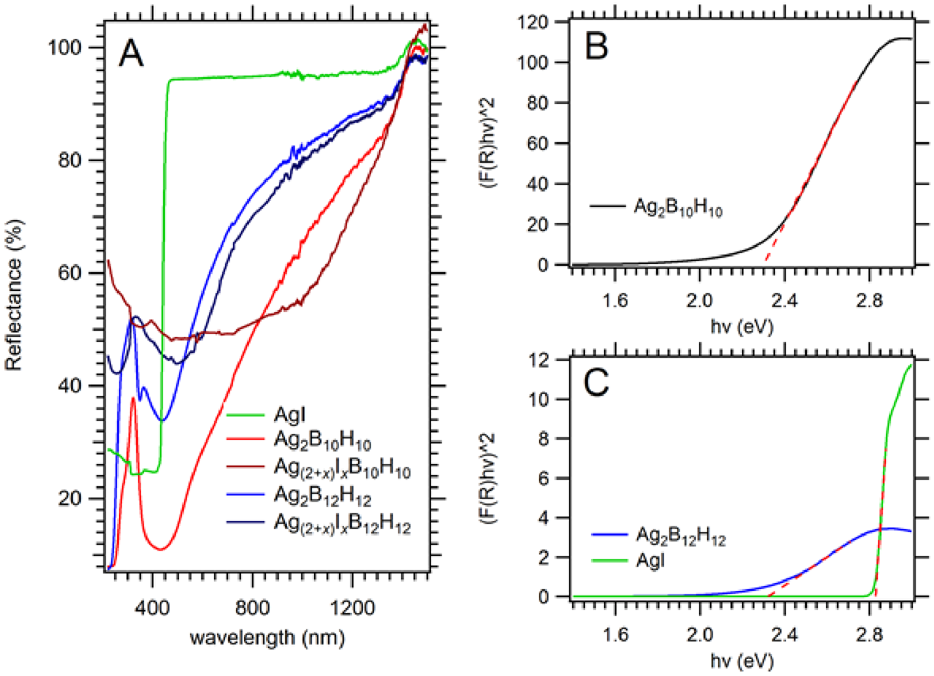

Supplementary Figure 10. A) UV-VIS spectra of silver *closo*-boranes in comparison to AgI. B) and C) show the direct bandgap extrapolations by the intersection of the red lines with the x-axis.

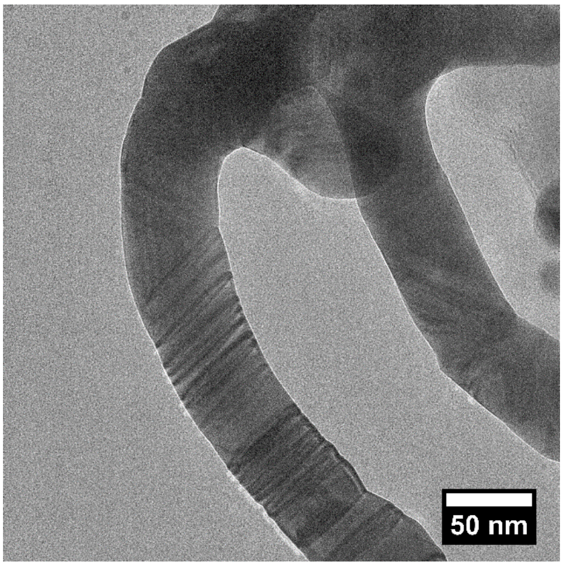

Supplementary Figure 11. Transmission Electron Micrograph (TEM) of Ag nanofilaments from $Ag_2B_{12}H_{12}$. Crystal twinning (dark bands) can be seen in the Ag filament that formed during growth.

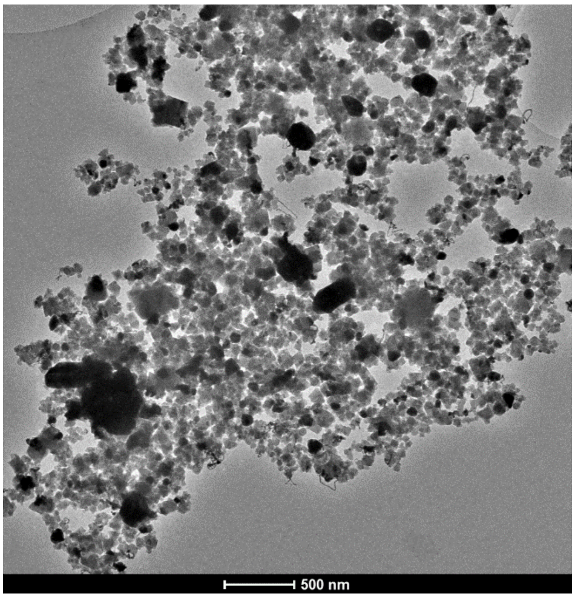

Supplementary Figure 12. Transmission Electron Micrograph (TEM) of thermally decomposed $Ag_2B_{12}H_{12}$ (to 400 °C) where dark particles are metallic Ag. The sample did not change under the electron beam in comparison to the pristine $Ag_2B_{12}H_{12}$, which formed Ag filaments.

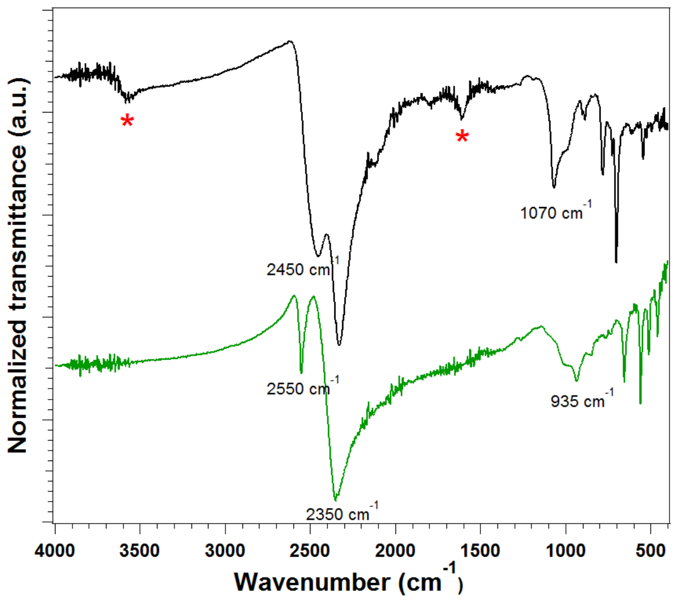

Supplementary Figure 13. Fourier Transform Infrared (FTIR) spectroscopy of dried $Ag_2B_{12}H_{12}$ (black line) and $Ag_2B_{10}H_{10}$ (green line) powders. Red asterisks indicate signal from O–H stretching and bending modes.

Supplementary Note 1. $Ag_{(2+x)}I_xB_{10}H_{10}$ and $Ag_{(2+x)}I_xB_{12}H_{12}$ crystal structures.

There are a number of issues that have so far prevented the structure solution of $Ag_{(2+x)}I_xB_{10}H_{10}$ and $Ag_{(2+x)}I_xB_{12}H_{12}$:

- Firstly, the new compounds have a small crystallite size and only exhibit 9 clear/unambiguous Bragg diffraction peaks over a *q*-range of 0.2 – 8 $Å^{-1}$, i.e. relatively large unit cell and relatively high symmetry.
- Secondly, the structures contain strong X-ray scatterers and absorbers, Ag and I, which may hamper localisation of light atoms like H and B.
- Thirdly, there are traces of reagents or decomposition products in the diffraction patterns that may overlap with minor peaks, thus making space group indexing particularly challenging in this case.
- It is also possible that the crystal structures of these highly conducting materials exhibit structural dynamics (i.e. cation partial occupancy and/or anion reorientation), which require high quality diffraction data, ideally on a single-phase compound.

Our preliminary research indicates that the unit cell of the new compounds, $Ag_{(2+x)}I_xB_{10}H_{10}$ and $Ag_{(2+x)}I_xB_{12}H_{12}$, could be large ~2400 $Å^3$. Unfortunately, with so few diffraction peaks and possible peak overlap with impurities, indexing such a large unit cell is currently unreliable.

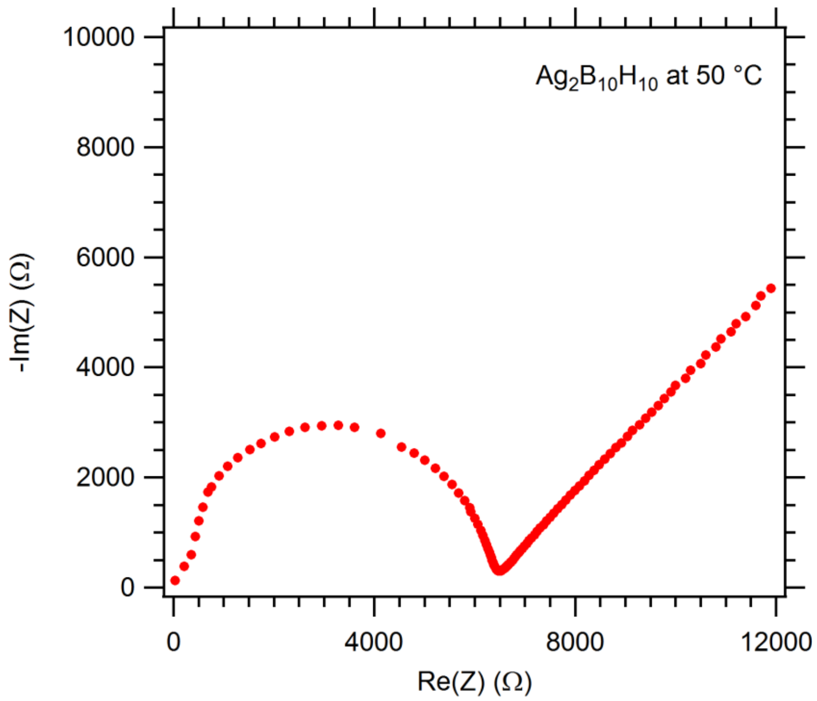

Supplementary Figure 14. Impedance spectrum (Nyquist Plot) for $Ag_2B_{10}H_{10}$ collected at 50 °C.
